# Efficacy of Hot Tea Infusion vs. Ethanolic Extract of *Moringa oleifera* for the Simultaneous Treatment of Nonalcoholic Fatty Liver, Hyperlipidemia, and Hyperglycemia in a Murine Model Fed with a High-Fat Diet

**DOI:** 10.1155/2024/2209581

**Published:** 2024-02-12

**Authors:** Salma I. Cortes-Alvarez, Ivan Delgado-Enciso, Alejandrina Rodriguez-Hernandez, Gustavo A. Hernandez-Fuentes, Nomely S. Aurelien-Cabezas, Norma A. Moy-Lopez, Nadia Y. Cortes-Alvarez, Jorge Guzman-Muñiz, Jose Guzman-Esquivel, Iram P. Rodriguez-Sanchez, Margarita L. Martinez-Fierro, Karen A. Mokay-Ramirez, Carlos E. Barajas-Saucedo, Carmen A. Sanchez-Ramirez

**Affiliations:** ^1^Department of Molecular Medicine and Nutrition Laboratory at School of Medicine, University of Colima, Colima, Colima, Mexico; ^2^Department of Research, Cancerology State Institute, Colima State Health Services, Colima, Colima, Mexico; ^3^Laboratory of Neuroscience, School of Psychology, University of Colima, Colima, Colima, Mexico; ^4^Department of Nursing and Midwifery, Division of Natural and Exact Sciences, University of Guanajuato, Guanajuato, Guanajuato, Mexico; ^5^Department of Research, Mexican Social Security Institute, Villa de Alvarez, Colima, Mexico; ^6^Molecular and Structural Physiology Laboratory, School of Biological Sciences, Autonomous University of Nuevo Leon, Monterrey, Nuevo Leon, Mexico; ^7^Molecular Medicine Laboratory, Academic Unit of Human Medicine and Health Sciences, Zacatecas Autonomous University, Zacatecas, Zacatecas, Mexico

## Abstract

*Moringa oleifera* (MO) is a native tree of Asia and is cultivated in some areas of Mexico as part of traditional horticulture. The aim of the present study was to compare the efficacy of MO infusion vs. MO ethanolic extract for the simultaneous treatment of nonalcoholic fatty liver (NAFLD), hyperlipidemia, and hyperglycemia in a murine model fed with a high-fat diet (HFD). BALB/c mice were fed a balanced diet (healthy control) or an HFD for 6 months. With this, the NAFLD model was established before starting a therapeutic intervention with MO for two months. The phytochemical analysis by nuclear magnetic resonance in ^1^H and ^13^C experiments showed signals for pyrrole alkaloids and triterpenes as the main constituents of the extract and infusion preparation. A significant reduction of SGPT, SGOT, lipids, urea, and glucose in blood among NAFLD groups treated with MO (infusion or extract) was found, when compared to the NAFLD-placebo group. Steatosis and liver inflammation were found to be decreased in the MO groups, as infusion or ethanolic extract. Infusion produced a better therapeutic effect than the extract in all parameters, except glycemic control, where the extract was better. As an additional finding, it is noteworthy that treatment with MO, particularly through infusion, resulted in improved motor activity. Moreover, a reduction in anxiety-like behavior was observed exclusively with the administration of infusion. These observations provide valuable insights into the potential broader effects of *Moringa oleifera* beyond the primary aim of the study.

## 1. Introduction

Westernized fast food has resulted in a surge in the consumption and accessibility of lipids, including high levels of saturated fats that can alter the eating habits of the population [1, 2]. The associations between the consumption of a high-lipid diet and diabetes, obesity, and liver disease, including nonalcoholic fatty liver disease (NAFLD), have long been recognized [3, 4]. The prevalence of NAFLD is up to 30% in developed countries and almost 10% in developing countries, making NAFLD the most common liver condition in the world [5].

NAFLD is the accumulation of hepatic steatosis not caused by excessive alcohol consumption. While it is often regarded as the hepatic manifestation of the metabolic syndrome, closely linked to conditions like obesity, insulin resistance, hyperlipidemia, and hypertension, this characterization has been a topic of ongoing discussion. Contrary to the general assumption, research indicates that around 30% of NAFLD patients do not exhibit symptoms of metabolic syndrome (MetS) [6]. Moreover, intriguingly, studies have shown that in many instances, NAFLD precedes the development of MetS [7–9]. Oxidative stress (OS) is considered an important factor in the pathophysiology and progression of chronic inflammatory liver diseases, including NAFLD [5, 10]. Therefore, the modulation of the antioxidant response emerges as an interesting target to prevent the development and progression of NAFLD. Given the absence of approved treatments by the Federal Drug Administration (FDA) to date, recent guidelines advocate for lifestyle interventions, bariatric surgery, and pharmacotherapy as potential options for managing nonalcoholic fatty liver disease (NAFLD). This encompasses the utilization of agents such as glucagon-like peptide-1 receptor agonists (GLP-1RAs), peroxisome proliferator-activated receptor-*γ* (PPAR-*γ*) agonists, and sodium-glucose cotransporter-2 (SGLT-2) inhibitors [11, 12].

Recently, there has been a remarkable progress in the field of herbal therapy due to the growing concerns about the development of drug resistance, drug side effects, and limited advances in the discovery of new drugs [13]. In almost all countries, medicinal plants have been widely used throughout history for the treatment of diseases as traditional healing remedies due to their broad therapeutic spectrum and minimal or no side effects [14]. Since there are no specific drugs available for NAFLD, considerable efforts have been focused on the search for new drugs and complementary/alternative medicines from different herbal formulations.

*Moringa oleifera* (MO) Lam. is a plant that belongs to the Moringaceae family, which is widely distributed in the tropics and subtropics and has been reported to possess various medicinal properties [15]. In Mexico, the use of *Moringa oleifera* is widely disseminated in the population, and even the government of the country considers *M. oleifera*, a plant species of high nutritional value, being used, almost entirely for human consumption [16, 17]. Ethnomedical studies refer to the use of *M. oleifera* tea infusion as follows: regulator of blood glucose levels, enhancer of the immune system, capable of regulating cholesterol levels, and with anti-inflammatory activity. Likewise, other ethnomedical precedents refer to the use of MO ethanol macerates to prevent and reduce hypertension [16, 17]. Phytochemical studies in MO have been shown to be a natural source of antioxidants such as phenolic compounds, vitamins A, C, and E, ascorbic acid oxidase, polyphenol oxidase, and catalase [18, 19]. Extracts from lyophilized leaves of MO have shown antioxidant and free radical scavenging activity [20–22]. Therefore, MO could be part of the therapeutic options for NAFLD.

Although MO is a plant currently under study, it has been suggested that the different stages of the plant and various extraction methods may influence its characteristics and potential effects [23], in addition to the fact that its effects are not always considered the same on mobility [24] and anxiety [25]. Therefore, the aim of the present study was to compare the therapeutic efficacy of hot tea infusion vs. MO ethanolic extract for the simultaneous treatments of nonalcoholic fatty liver, hyperlipidemia, and hyperglycemia in a murine model fed with a high-lipid diet with NAFLD.

## 2. Materials and Methods

### 2.1. Plant Material

The fresh leaves of MO were collected in the city of Colima, Mexico (9.2433°N latitude and 103.7242°W longitude). The plant material was taxonomically identified and compared with a specimen from the National Herbarium of Mexico (MEXU), with voucher number MEXU: 1115057 serving as the reference. Subsequently, the leaves were conditioned in the laboratory, thoroughly cleaned of extraneous materials, and dried in an oven with air circulation at 37°C for 60 hours.

### 2.2. Preparation of the Infusion

The dried leaves of MO were finely crushed using a mortar and pestle, achieving a loose tea-like consistency. Daily infusions were prepared, combining 0.7 g of MO leaves with 10 mL of distilled/deionized water. The water was heated to boiling point (95–100°C), then poured over the dried MO leaves, and stirred for 15 minutes. Following this, the infusion was filtered through cotton gauze to remove solids. The resulting filtrate (MO infusion tea) was allowed to cool to body temperature before administration. This method adheres to the traditional practices of Colima, Mexico, ensuring the proper preparation of the MO leaf infusion [16, 17].

### 2.3. Preparation of the Ethanol Extract

According to Mousa et al., ethanolic extract was prepared from MO-dried leaves [26]. The cold extraction process was employed to obtain the crude extract. In this process, 1 kg of the powdered material was combined with 2000 mL of ethanol (96%). The mixture was allowed to macerate for 72 hours at room temperature (22°C), with intermittent gentle shaking. Following maceration, the mixture was filtered sequentially through cotton gauze and Whatman filter paper No. 1, resulting in a green-hued solution. To concentrate the solution, an initial step involved using a vacuum rotary evaporator under specific conditions (adjustment bath: 40°C, rotation: 50 rpm, pressure: ∼15 psi, and condenser: 4°C) to remove ethanol. The solution was subsequently further concentrated through freeze-drying using a freeze dryer. The yield of the extract was determined in % (w/w). The MO ethanol extract was stored within a silicon desiccator until its subsequent utilization.

### 2.4. Phytochemical Screening of the MO Infusion and Ethanol Extract

A small portion of the dry ethanolic extract or a tea infusion of *M. oleifera* leaf was utilized for the phytochemical tests, including tannins, flavonoids, alkaloids, saponins, and steroids, following established methods with some modifications [27].

In the preliminary phytochemical analysis of the ethanol extract, a solution was prepared by dissolving 10 mg of the ethanolic extract in the solvent indicated by the specific test. Regarding the infusion, 1 ml of the infusion filtrate used for the tests (see preparing infusion section) was used. For the tannin test, 1 ml of plant solution extract or infusion filtrate was mixed with 5 ml of distilled water and filtered (using Whatman No. 1 filter paper). The presence of tannins was indicated by a blue coloration resulting from the addition of ferric chloride reagent to the filtrate. Alkaloid presence was determined by Dragendorff's test; 10 mg of the plant extract or 1 ml of infusion filtrate was dispersed in 5 ml of 1% HCl in a steam bath. A milliliter of the mixture was treated with few drops of Dragendorff's reagent; a positive reaction was considered if a precipitate or turbidity was formed. The occurrence of a red or orange coloration was indicative of the flavonoids in the presence of HCl and magnesium. A freshly prepared 7% blood agar plate was used, and wells were made in it. The crude extract (10 mg) or infusion filtrate (1 ml) was mixed with 5 ml of a 9 : 1 water/methanol solution and subsequently used to fill the wells bored in the blood agar plates. 9 : 1 water/methanol solution was used as a negative control, while commercial saponin solution was used as a positive control. The plates were incubated at 35°C for 6 h. A complete hemolysis test of blood around the extract was indicative of saponin. Steroid rings were indicated by the reaction with H_2_SO_4_ in chloroform solution. The reddish-brown color on the interface was taken as positive for steroid rings [27].

### 2.5. Quantitative Determination of Flavonoids, Antioxidant Capacity, and Reducing Power of MO Infusion and Ethanol Extract

To achieve a comprehensive screening and quantification of flavonoid compounds, along with assessing the antioxidant capacity and reducing power of the *Moringa oleifera* (MO) infusion and ethanol extract, additional assays were conducted. For the MO infusion tea, a 1.0 ml sample of the infusion filtrate was pretreated by lyophilization using an ILShinBioBase freeze dryer (model TFD8503, Korea). This lyophilization process effectively removed the water content, maintaining the constituents of the infusion in a dry state. The resulting lyophilized powder was stored in an ultracooler at −40°C until analysis. For both the MO extract and the lyophilized infusion, the working concentration was adjusted to 5 mg/ml for subsequent analysis.

The analysis involved the determination of total flavonoid content (TFC), expressed as quercetin equivalent in *μ*g/mg of extract, using the method by Wakeel et al., 2019 [28]. In addition, total antioxidant capacity (TAC) was assessed through the phosphomolybdenum method [29, 30], using ascorbic acid as the positive control. The calculation of antioxidant capacity followed the formula: % antioxidant capacity = [1 − (OD of the sample/OD of the control)] *∗* 100. Furthermore, the ferric reducing power assay (FRPA) was conducted using the potassium ferricyanide-ferrous chloride method [31, 32], with modifications from Wakeel et al., 2019. The reducing power was calculated as % reducing power = [1 − (OD of the sample/OD of the control)] *∗* 100. All the determinations were carried out using a BioMate THERMO spectrophotometer (USA).

### 2.6. Nuclear Magnetic Resonance General Screening

In order to obtain a general screening of the compounds in the MO infusion and ethanolic extract that used a nuclear magnetic resonance (NMR) (^1^H and ^13^C), spectra were obtained using a Bruker NMR spectrometer (Bruker, Leipzig, Germany), with an operating frequency of 400 and 100 MHz, respectively. For the *Moringa oleifera* (MO) infusion tea, a sample pretreatment was conducted by lyophilizing an infusion sample using an ILShinBioBase freeze dryer (model TFD8503, Korea). It is important to note that a larger quantity of the infusion sample was lyophilized for analysis, (30 ml of infusion filtrate) following the conditions described above. In addition, the ethanol extract was directly prepared for analysis using the extract obtained previously. Both the MO-lyophilized infusion and the ethanol extract were analyzed at a concentration of 10 mg/ml for consistency in testing conditions employing CDCl_3_ (Chloroform-d, Sigma Aldrich, USA) as a solvent. The chemical shifts are given in *δ* (ppm), and coupling constants (J) are reported in Hz. The chemical shifts obtained were compared with some of the isolated compounds from *M. oleifera* leaves (see S2 Table).

### 2.7. Experimental Animals

**Figure 1:**
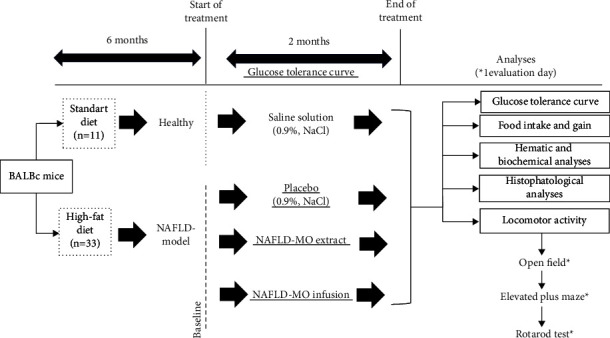
General diagram of the research. BALB/c mice were fed a balanced diet (healthy control) or a high-fat diet for 6 months. With this, the NAFLD model was established before starting a therapeutic intervention with MO for 2 months.

The research was conducted as a prospective, single-blind, 4-arm, parallel-group, randomized, preclinical trial, adhering to the guidelines outlined in the “ARRIVE Essential 10” for Animal Research (see Figure 1) [33]. Blinding consisted of the evaluators not knowing the group to which the mice or samples analyzed belonged (pathologist, evaluator of blood, and biochemical, motor, or behavioral tests) [33]. The calculation of the sample size in this study was based on the formula derived from previous reports, which takes into account the assessment of incidence [34] and uses the reduction of nonalcoholic fatty liver disease (NAFLD) as the primary outcome measure [35]. It was determined that a minimum of nine animals per group was necessary to establish meaningful comparisons. A total of 44 mice were randomly assigned to four groups for the purpose of this study.

This experimental study included 44 BALB/c mice (Envigo®, Mexico) between 4 and 6 weeks old with an initial weight of 22 to 25 g. The 44 mice were randomly assigned into two groups: a healthy group (*n* = 11, consisting of 6 males and 5 females) and an NAFLD experimental group (*n* = 33, comprising 18 males and 15 females). The mice were kept in cages, with a maximum of 5 mice per group. Conditions were controlled (12 h: 12 h light/dark cycle, 23°C temperature with 50% humidity), and the animals had access to food and water ad libitum. 11 mice were fed with a standard diet made up of 44.2% carbohydrates, 18.6% protein, 3.5% fiber, and 6.2% net fat (2018S Teklan and Global 18% Protein Rodent Diet, Envigo®, USA) and therefore were considered the normality reference (healthy group). All mice were placed with a metal tag with a unique number on the pinna of the ear, which allowed correct identification during the experiment. For the induction of the NAFLD model, 33 mice were fed with a high-fat diet (HFD) that was made up of 46.9% carbohydrates, 17.3% protein, 25% net fat, 1.25% cholesterol, and 0.5% cholic acid [35]. The model used to generate nonalcoholic fatty liver (NAFLD) requires 6 months of exposure to a high-fat diet to express pathophysiological alterations similar to the progression of the disease in humans and to facilitate the examination of long-term treatments and their effects [36]. After 6 months of administering the aforementioned diets (with the established NAFLD model), HFD-fed mice were randomly divided into three groups, of which two groups were given therapeutic treatment with MO (NAFLD-MO infusion and NAFLD-MO ethanolic extract groups), leaving one group without treatment (the NAFLD-placebo group, with administration of saline solution), which was the point of reference for the pathologic alterations caused by the model. Randomization was performed using computer-generated random allocation cards. The treatments were administered for 60 days (from the sixth to the eighth month). At the end of the treatment, a glucose tolerance curve and psychomotor analysis were performed. After the completion of the treatment period, euthanasia was performed on all animals twenty-four hours later. Trained research personnel conducted euthanasia in accordance with the American Veterinary Medical Association's (AVMA) Guidelines for Euthanasia of Animals: 2020 Edition. Prior to euthanasia, mice were anesthetized with intraperitoneal administration of sodium pentobarbital at a dose of 50 mg/kg. Manual cervical dislocation was then carried out, involving the placement of the thumb and forefinger on either side of the neck at the base of the skull, just behind the ears, while keeping the head still. Simultaneously, the other hand pulled on the base of the tail with a firm, steady motion, causing the cervical vertebrae to detach from the skull [37]. Subsequently, blood samples were collected by cardiac puncture for further biochemical analyses. The weight of mice in all groups ranged from 21.2 to 38.3 grams, within the allowable weight for euthanasia by cervical dislocation [37]. The livers were promptly excised, weighed, and subjected to histopathological examination, as illustrated in Figure 1. In addition, the right kidney, left kidney, pancreas, brain, ovaries, and epididymal fat pad were obtained and weighed. Trial protocols were approved by Colima State Cancer Institute's Research Ethics Committee, Mexico (Protocol Number: CEICC-240818-ETAMORI-010), which ensured compliance with national and international legal and ethical requirements. Animals were handled in accordance with institutional guidelines, the Mexican official norm governing laboratory animal use (NOM-062-ZOO-1999) and the National Academy of Science's (2011) Guide for the Care and Use of Laboratory Animals.

This experimental study included four groups: (1) healthy group; (2) NAFLD-placebo (saline solution); (3) NAFLD with treatment of ethanolic extract of MO; and (4) NAFLD treatment with infusion of MO. After two months of treatment, blood parameters and the liver tissues were analyzed. Biochemical, histological, and psychomotor analyzes were performed.

### 2.8. Dose Calculation

Based on the traditional community use, the MO infusion is usually prepared with approximately 3 g of dried leaves in 100 ml of water, which is generally consumed by an adult with a considered average weight of 70 kg. This is equivalent to a usual human dose of 42.85 mg/kg. With these data, the dose for mice was calculated using the US FDA recommendations for dose extrapolation between species [38]. The equivalent mouse dose was 500 mg/kg/day in 200 *μ*l volume for both extraction systems (infusion and ethanol), determined according to what was reported in the literature as an optimal dose served [39]. Administration of all groups of treated or untreated mice was by an orogastric route using saline solution as the vehicle.

### 2.9. Body Weight and Food Intake

The body weight (g) was recorded since day one and then weekly consecutively for eight months using an automated electronic scale (A & D Weighing, Limited HR-200, CA, USA). To weigh the mice, a round plastic container was placed on the scale and tarred to zero before placing the mouse inside the container. In addition to this, the daily food intake for each group was measured weekly for eight months. Food intake was estimated as follows: [total food consumed per cage]/[mice per cage] × [days of food consumption]. The data are expressed as the mean ± standard deviation or standard error of the mean.

### 2.10. Hematic Biometry and Biochemical Profile Analysis

A sample of blood was obtained by cardiac puncture after euthanization. The samples were collected and deposited in vacutainer tubes without anticoagulant (EDTA) allowing blood to clot. The blood without EDTA was processed in a Beckman Coulter AC-T instrument (Beckman Coulter, Inc., Brea, CA, USA), and the following blood parameters were estimated: erythrocytes, leukocytes, platelets, hemoglobin, and hematocrit. The blood without anticoagulant was centrifuged at 3500 rpm for 5 minutes to obtain serum, which was immediately transferred with a pipette to the allocated containers and was determined using an automatic biochemical analyzer (Cobas c111, Roche®, Mexico). The serum was analyzed for the estimation of the following biochemical parameters: serum alanine aminotransferase (SGPT), aspartate aminotransferase (SGOT), cholesterol, triglycerides (TG), total lipids, urea, and glucose.

### 2.11. Glucose Tolerance Curve (OGTT)

Fasting glucose was determined at months 0, 3, and 6 (before starting the treatments). An intraperitoneal glucose tolerance test was carried out at the 6^th^ month (before starting treatment) and 8^th^ month (after 2 months of treatment), as described by Vinué and González-Navarro [40]. The animals were made to fast for a period of 6 hours. The blood was collected from the tail vein of each mouse (20 *μ*l), and before initiating the tests, 0-minute blood samples were withdrawn to estimate fasting glucose levels. The intraperitoneal injection of glucose at a dosage of 2 mg/kg was performed, and the levels of blood glucose were measured at intervals of 0, 30, 60, 90, and 120 minutes following administration. The area under curve (AUC) was calculated by using the trapezoid rule [41].

### 2.12. Histopathologic Liver Analysis

The liver tissues were fixed, processed, embedded in paraffin wax, sectioned (5 *μ*m thick), stained (hematoxylin/eosin), and analyzed on digital images of the entire surface of each liver sample (right and left lobules) as previously described [42]. A pathologist conducted a blinded evaluation of all histological parameters. Hepatic steatosis was classified according to the percentage of liver tissue that presented with fat accumulation, which was grade 0 (absent), grade 1 or mild (up to 33%), grade 2 or moderate (between 33 and 66%), and grade 3 or severe (more than 66%) [36]. Inflammation was evaluated by functioning histologic zones depending on the oxygen supply as previously described, being classified into four categories according to the percentage of tissue presenting with inflammatory infiltrate: none (0%), mild (1 to 32%), moderate 33–66%), and severe (>66%) [42, 43].

### 2.13. Locomotor Activity Evaluation

The psychomotor analysis experiments are crucial for evaluating the potential impact of *Moringa oleifera* treatment on both anxiety-related behavior and muscular tone in mice. These assessments will provide a comprehensive understanding of the treatment's effects, aligning with the goal of exploring the broader implications of *Moringa oleifera* in the context of nonalcoholic fatty liver disease (NAFLD).

### 2.14. Open Field

Following the treatment, mice were evaluated for locomotion impairment using an open field activity monitoring system, which is a useful tool for studying locomotion impairment in animal models [44]. This test was carried out as previously described by Montes-Galindo et al., 2019 [42], considering an alteration in the locomotor activity in case a significant change occurred in the speed of the experimental group, compared to that of the control group [45].

### 2.15. Elevated Plus Maze

A widely used rodent behavioral assay called the elevated plus maze has been validated for assessing the antianxiety effects of medications [46]. This evaluation was carried out following previously reported specifications [47], calculating the anxiety-like behavior index according to Cohen et al. [48].

### 2.16. Rotarod Test

When the treatments were finished the mice were trained on 3 consecutive days on a rotarod (LE8300; Letica LSI, Pan-lab Scientific Instruments, Barcelona, Spain) at a speed of 18 rotations per minute. We recorded rotations and falls. These variables were used to identify any alterations in motor coordination and balance [49].

### 2.17. Statistical Analysis

For descriptive statistics, the data were represented as the mean ± standard deviation, or, in certain cases, utilizing the standard error of the mean (SEM). The normal distribution of the data was evaluated through the application of the Kolmogorov–Smirnov test. Subsequently, the equality of variances was verified using Levene's test. To ascertain differences between groups, one-way ANOVA was performed, followed by Tukey's test post hoc analysis. The statistical analyses were conducted using IBM SPSS version 20 software (IBM SPSS, Chicago, Illinois, USA). A *p* value of <0.05 was used to determine statistical significance.

## 3. Results

The resultant yield of the ethanolic extract of MO was determined to be 3.5% w/w. The phytochemical analysis (Table 1) carried out on the ethanolic extract of the MO leaf extract revealed the presence of tannins, alkaloids, steroids, and saponins. On the other hand, in the aqueous infusion, it was possible to detect flavonoids, steroids, saponins, and a slight presence of alkaloids. These compounds represent some of the prominent metabolite families within the plant species. However, it is important to note that the list provided is not exhaustive, and the presence of other phytochemicals cannot be ruled out. Other compounds not included in the analysis might also contribute to potential pharmacological activities, but further studies are necessary.

The total flavonoid content in *M. oleifera* samples was quantified using a standard quercetin calibration curve (*y* = 0.0027*x* + 0.0058, *R*^2^ = 0.9986). The results are expressed in Table 1 as the total flavonoid content (TFC) in *μ*g of quercetin equivalents (QE)/mg by extract. The highest flavonoid content was quantified in the infusion tea, reaching 172.66 ± 7.58 *μ*g/mg extract, while the ethanolic extract showed a concentration of 19.40 ± 10.68 *μ*g/mg. The reduction of ferricyanide (Fe^3+^) to ferrous state (Fe^2+^) by plant phytochemicals is a good indicator of antioxidant activity [50]. In this study, the percentage (%) of reducing power of the ethanol extract of *M. oleifera* was estimated against the standard drug ascorbic acid (Table 1). The reducing capacity of both *Moringa* samples (extract and infusion) showed values near that of ascorbic acid. In the case of the antioxidant activity in comparison to the ascorbic acid, both samples show a near 50% activity. In particular, the ethanol extract of *Moringa* slightly surpasses the infusion by around 10%.

^1^H and ^13^C NMR spectra as shown in Figures S1–S4 were obtained for the evaluated extracts (MO EtOH and infusion). Signals in the spectra were assigned, and the shifts were compared to isolated compounds from *M. oleifera* (S1 Table). Regarding the extract, it could be hypothesized that it is rich in alkaloid-type compounds. In these extracts, some of the signals obtained in ^1^H and ^13^C are like those reported for niazirin and also similar to niazirinin; alkaloids were identified from MO leaves [51, 52]. Both the extract and the infusion presented signals around 7.05 to 9.03 ppm, like what was expected for this type of alkaloids. In respect to the MO infusion, most of the signals assigned are found in the ethanolic extract but in smaller intensity (125 to 140 ppm). The comparison with the literature allowed us to compare some triterpene molecules, such as *β*-sitosterol [53], finding that this possible presence could explain the positive reaction with the sulfuric acid result obtained in the preliminary phytochemical screening. Based on the comparison of the NMR spectra and the phytochemical background of the species, it is suggested that the MO extract and infusion evaluated in these experiments are mostly composed of alkaloids, triterpenes, and flavonoids, which are glycosylated evidenced by signals around 3.0–5.0 ppm.

### 3.1. Food Intake and Weight Gain

Regarding food consumption, throughout the period from the fourth to the sixth month (5.32 ± 0.6 in healthy vs. 7.40 ± 0.3 in the NAFLD model for the fourth month and 6.17 ± 1.2 in healthy vs. 8.15 ± 0.2 in the NAFLD model for the sixth month), it was consistently found that the mice in the NAFLD model presented a significantly higher consumption than those in the healthy group (*p* < 0.001 for all periods).

**Figure 2:**
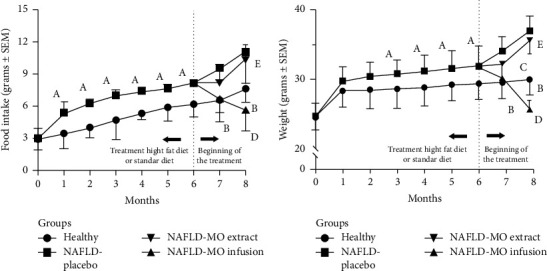
Food intake and body weight evolution in the groups. (a) Food intake; (b) body weight evolution. All values are expressed as mean ± SEM, (*n* = 11); the differences of Tukey's post hoc analysis are marked when they are significant: ^A^*p* ≤ 0.05, healthy vs. NAFLD model genesis, ^B^*p* ≤ 0.05, healthy vs. NAFLD-placebo, ^C^*p* ≤ 0.05, healthy vs. NAFLD-MO infusion, ^D^*p* ≤ 0.05, NAFLD-placebo vs. NAFLD-MO infusion, and ^E^*p* ≤ 0.05, NAFLD-MO extract vs. NAFLD-MO infusion. The comparisons between NAFLD-placebo vs. NAFLD-MO extract and healthy vs. NAFLD-MO extract did not show statistically significant differences at a significance level of *p* ≤ 0.05.

Once the treatments started (sixth month), during 2 months that MO was administered, it was observed that in the last month, the NAFLD-MO infusion group consumed significantly less food than the NAFLD-placebo group (5.64 ± 2.0 g vs. 11.0 ± 2.9 g, *p*=0.020), and when comparing between the group treated with NAFLD-MO infusion vs. NAFLD-MO ethanolic extract, it was found that the group that received infusion consumed significantly less food than the group that received extract (5.64 ± 2.0 g vs. 10.27 ± 1.4 g, *p*=0.010) (see Figure 2(a) and SII Table).

Regarding the weight gain from the third month until the end of the treatments, there was significantly greater weight gain in the NAFLD-placebo vs. the healthy group (36.92 ± 2.1 vs. 29.95 ± 2.2, *p* ≤ 0.001). In the last month of the treatment, less weight gain was observed only in the NAFLD-MO infusion group compared to the NAFLD-placebo group (25.70 ± 1.2 g vs. 36.92 ± 2.1 g, respectively, *p* ≤ 0.001), as well as to the NAFLD-MO ethanolic extract (25.70 ± 1.2 g vs. 35.53 ± 1.9 g, respectively, *p* ≤ 0.001), and even with the healthy group (25.70 ± 1.2 g vs. 29.95 ± 2.2 g, respectively, *p* ≤ 0.001), see Figure 2(b) and SII Table.

### 3.2. Biochemical and Hematological Parameters

A significant decrease in cholesterol, triglycerides, total lipids, SGOT, SGPT, and glucose was found in the NAFLD groups treated with MO infusion and in ethanolic extract compared to the NAFLD-placebo group (*p*=0.001). When doing the comparison between the group treated with infusion vs. ethanolic extract, lower levels of triglycerides SGOT and SGPT were found in the group that received infusion vs. the ethanolic extract (*p*=0.020). On the other hand, regarding the hematic parameters, only significantly higher hemoglobin levels were found in the group treated with infusion than in the healthy control group (*p*=0.001), as shown in Table 2.

### 3.3. Glucose Tolerance Curve

With the aim of ensuring the comprehensive monitoring of individuals throughout the study, observations were made at months 0, 3, and 6. Basal fasting glucose levels were comparable across all groups at the study's onset (*p*=0.153). The influence of a high-fat diet on hyperglycemia became evident after three months, as evident when compared with the standard diet (Table 3). Before the commencement of any treatments, a glucose tolerance test was conducted at the 6-month mark. This assessment highlighted that the healthy group displayed significantly lower glucose levels (across all measurement intervals: basal, 0, 30, 60, 90, and 120 minutes) when contrasted with all NAFLD-afflicted groups (*p* < 0.001).

**Figure 3:**
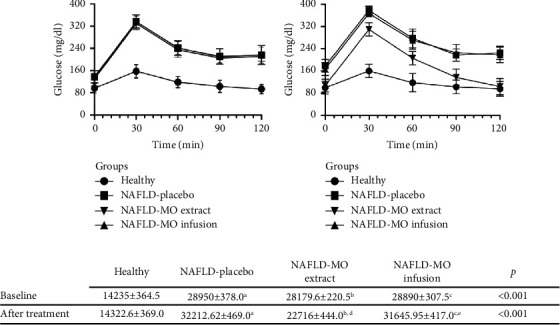
Glucose tolerance curve in the groups. (a) Baseline: before starting the treatments, the healthy group obtained significantly lower glucose levels (at all measurement times, basal, 0, 30, 60, 90, and 120 minutes compared to all NAFLD groups (*p* < 0.001). (b) After treatment: at the end of the treatment with MO. (c) AUCs of glucose levels (mg^.^min/dl) calculated according to trapezoid rules with those from OGTT. All values are expressed as mean ± SEM, and differences from paired *t*-test analysis are marked when they are significant: ^A^*p* < 0.05, healthy vs. NAFLD-placebo, ^B^*p* < 0.05, healthy vs. NAFLD-MO ethanolic extract, ^C^*p* < 0.05, healthy vs. NAFLD-MO infusion, ^D^*p* < 0.05, NAFLD-placebo vs. NAFLD-MO ethanolic extract, and ^E^*p* < 0.05, NAFLD-MO ethanolic extract vs. NAFLD-MO infusion. *n* = 11 per group.

This background context sets the stage for understanding the subsequent results. Considering these observations, the MO treatment displayed reductions in glucose levels within the NAFLD-MO ethanolic extract group, establishing its superiority over the NAFLD-placebo group (*p*=0.010). Moreover, a direct comparison between the NAFLD-MO infusion group and the NAFLD-MO ethanolic extract group highlighted consistently lower glucose levels in the latter (*p*=0.001), as detailed in SIII Table and visually depicted in Figure 3.

### 3.4. Organ's Weight

The measurements of organ weights, including the liver, right kidney, left kidney, pancreas, brain, and ovaries, as well as the epididymal fat pad, are detailed in Table 4. This comprehensive evaluation was conducted as part of the study's effort to provide insights into the potential safety implications of MO. Notably, a significantly lower liver weight was found in the group treated with infusion compared to the NAFLD-placebo group (*p*=0.048), and a corresponding significant reduction in epididymal fat pad was also observed (*p*=0.001), as indicated in Table 4.

### 3.5. Histopathological Parameters

**Figure 4:**
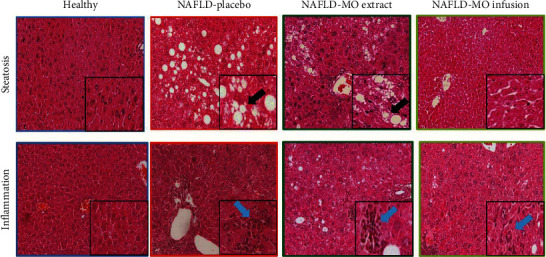
Histopathological parameters in the groups. It is possible to observe that the liver tissues in the standard diet (SD) group do not show pathological changes. The NAFLD-placebo group shows a greater presence of signs of steatosis and inflammation than the liver tissues of the groups treated with NAFLD-MO. As an example, the black arrow shows an area of steatosis, while the blue arrow shows the presence of inflammatory cells. Microphotographs stained with hematoxylin and eosin at xx magnification, with the framed area presumably at ×200 magnification.

Regarding steatosis and inflammation, significantly lower percentages were found in the two NAFLD groups treated with MO than in the NAFLD-placebo group (*p* ≤ 0.001). When making the comparison between the NAFLD groups treated with MO, significantly lower percentages of steatosis and inflammation were observed in the group that received the infusion than in the group treated with the ethanolic extract (*p*=0.002), as can be seen in Table 5 and Figure 4.

### 3.6. Locomotor Activity Evaluation

To evaluate mobility strength, we measured the number of rotations and turns. Regarding the number of rotations, a significantly higher number was observed in the NAFLD group treated with infusion than in the SD diet and the NAFLD-placebo group (*p*=0.003). On the other hand, regarding the number of falls, a significantly lower number of falls were observed in the NAFLD groups treated with MO in relation to NAFLD with placebo (*p* < 0.001). Finally, the anxiety-like behavior index was calculated in the elevated plus-maze experiment. In this experiment, the anxiety-like behavior index is referred to as the number of crossings of the mouse between the open and closed arms, as well as, the time that it remained in each of the arms of the maze. In our results, a lower anxiety-like behavior index was found in the NAFLD group treated with MO in the form of infusion than in the NAFLD-placebo group, and even to the healthy group (*p* < 0.001). When making the comparison between infusion vs. ethanolic extract, the group with infusion showed a lower rate of anxiety-like behavior (*p* < 0.001), as shown in Table 6.

## 4. Discussion

The objective of the present study was to compare the efficacy of hot tea infusion vs. ethanolic extract of MO for the simultaneous treatment of nonalcoholic fatty liver, hyperlipidemia, and hyperglycemia in a murine model fed with a high-lipid diet and an established NAFLD. An important point to discuss is that the same amount of ethanolic extract was used as the leaf used for the infusion (traditional form) [16, 17]. Although the concentration of metabolites would be different, the objective of the study is to present a comparison of the response to exposure of *Moringa oleifera* as a reflection about what the population consumes. However, future studies are required to clarify the concentration of metabolites consumed by the individual in an infusion and in an ethanolic preparation.

First, the findings showed that the administration of MO in both infusion and ethanolic extract in individuals with NAFLD reduced food intake, when compared with the NAFLD group with placebo at the seventh month. This result coincides with previous studies, which have identified that the administration of ethanolic extract for 49 days [51] and methanolic extract of MO to mice fed with HFD reduced the food consumption, even, as early as, 21 days after beginning the extract administration [52]. In the same way, Rojas-Silva found that the cumulative food intake was lower in the group of mice fed with an HFD +5% MO concentrate from 12 week [53].

It is important to highlight that in the group treated with the infusion of MO, the food intake of mice was significantly reduced (from the seventh to the end of the experiment), simultaneously leading to weight loss (see Figure 2) and improvement in NAFLD (see Figure 4). These phenomena could be related to the expression of anorexigenic peptides, both of hypothalamic and intestinal origin, which play an important role in the pathology of NAFLD [54, 55] and may be modulated by the metabolites present in the infusion (see Table 1). However, further experiments are necessary to identify and isolate the specific metabolites present, elucidating their mechanisms of action.

Second, the present study identified a decrease in the levels of total cholesterol, triglycerides, total lipids, SGOT, SGPT, and glucose in the groups treated with MO, in both infusion and ethanolic extracts, compared to the NAFLD group without treatment. This finding supports earlier reports that have suggested that MO at different doses lowers total cholesterol [56, 57], triglycerides [58], ALT, and AST [59] in rats fed with HFD + MO in different doses.

Figure 4 shows that the mice treated with the ethanolic extract of MO presented better glycemic control than the mice treated with the infusion. This effect could be due to differences in the composition and abundance of the secondary metabolites (alkaloids). Some previous phytochemical studies indicate that high polarity extracts (ethanol and methanol) [60] are abundant in pyrrole-type glycosylated alkaloids (niazirin, niazirinin, marumoside A, marumoside B, 4-((4′-0-acetyl-a-L-rhamnosyloxy) benzyl) isothiocyanate, and pyrrolemarumin-4″-O-*α*-L-rhamnopyranoside) [61, 62]. These high polarity extracts have shown antidiabetic activity, anti-inflammatory capacity, and radical scavenging activity [63]. It is important to point out that, in the preliminary phytochemical tests, the ethanolic extract showed a more intense response to the alkaloid assay, which could probably explain its better effect on glycemia management. However, more studies are required to identify, isolate, and evaluate these compounds, as well as the molecular mechanisms involved in this phenomenon.

Previous research has revealed that cholesterol decrease is possibly attributable to the presence of bioactive phytoconstituents, known as ∂- and *β*-sitosterols [60, 61] that are known for reducing the level of LDL-c in serum without modifying the high-density lipoproteins or triglycerides [60, 63]. Another suggested reason why MO exerts hypolipidemic effects is chlorogenic acid (CGA), since it is one of the bioactive components present in sufficient quantities in MO [64]. Although the exact phytoconstituent responsible for the effects observed in this study is not yet known, studies have shown that many of these phytochemicals possess beneficial effects, such as stimulation of glucose transport, inhibition of adipocyte differentiation, lowering of ALT, AST, total cholesterol, and triglycerides [65], and the attenuation of oxidative stress [57].

Third, one of the indicators used to identify the therapeutic effect of MO on NAFLD has been hepatic steatosis; our results showed a quantitative-qualitative decrease in steatosis after the administration of MO, in infusion or ethanolic extract. The effect of MO on steatosis has been reported in some studies. For example, Faizi et al., after feeding rats with an HFD for 49 days, observed a development of a high degree of steatosis in the rats; however, the group exposed to the HFD plus daily supplementation of 400 mg/kg of methanolic extract of MO showed a decrease in the development of hepatic steatosis [52]. Similarly, another study using MO at a high concentration (15%) showed a lower degree of steatosis in animals fed an atherogenic diet. Finally, MO has been associated with hepatoprotective effects not only in response to the consumption of unbalanced diets but also in mitigating potential liver damage by drug-induced toxicity [66, 67]. Despite the evidence of the positive effect of MO on hepatocytes, the mechanisms involved have not been fully elucidated. The literature suggests that MO may prevent hepatic steatosis by affecting gene expression related to hepatic lipid synthesis, resulting in lower cholesterol and triglyceride concentrations and reduced inflammation in the liver [68]. Quercetin and chlorogenic acid have demonstrated the ability to reduce triglyceride by inhibiting diacylglycerol acyltransferase-2 (DGAT2) [68], a key microsomal enzyme involved in the biosynthesis of TG. Given that MO is rich in these compounds [64], it is speculated that their presence in MO may contribute to decrease DGAT2 expression, potentially explaining the observed reduction in triglyceride concentrations in animal models with MO administration [68].

Likewise, it is presumed that the therapeutic effects of MO may be attributable to characteristic phytochemicals, glucosinolates, and isothiocyanates, found in many parts of the MO tree and quite prominently in its leaves, which participate in the elimination of reactive oxygen species and cellular redox balance [20, 69].

Fourth, the second and last histological indicator to identify the therapeutic effect of MO on NAFLD was hepatic inflammation. The effect of MO on inflammation has been studied to a lesser extent. Among the findings, there are those that showed that chronic exposure to an HFD + supplementation of methanolic extract of MO prevented the formation of hepatic inflammation compared to nonsupplemented animals, who developed a higher degree of inflammation [51].

On the other hand, Hamza et al. confirmed the hepatoprotective properties by the marked improvement in the necroinflammatory score in the animals that received MO compared to their counterparts who did not [70, 71]. Joung et al. demonstrated the suppression of inflammation caused by a high-lipid diet with the administration of MO [72]. These results suggest that MO has hepatoprotective and anti-inflammatory properties against liver damage, due to its antioxidant properties and anti-inflammatory effects [69].

The results showed a significantly greater effect on hepatic steatosis and inflammation and biochemical values of ALT, AST, total cholesterol, and triglycerides when the administration of MO was in infusion. Tea remains one of the most frequently consumed beverages, on account of its antioxidant or pro-oxidant properties. Active compounds in tea, particularly tea polyphenols, can directly or indirectly scavenge ROS to reduce oncogenesis, cancer, and metastasis [73, 74]. However, it is possible that the combination of polar phenolic compounds like flavonoids and tannins with alkaloids is responsible for this effect. Preliminary analysis of the plant's chemical composition showed that both the ethanolic extract and the hot water infusion have similar profiles to the tested secondary compounds, except for flavonoids (less concentration in MO ethanolic extract than in infusion). Flavonoids were found in the hot water infusion, likely due to higher extractability caused by heat. There was also a slight difference in precipitate formation observed during alkaloid selection. Therefore, it can be concluded that the slight difference between the two is due to the variation in phytoconstituent content, which could be caused by differences in active ingredient concentration or the decomposition of active ingredients during the preparation of the hot tea infusion using gentle heat [75]. This could explain the better results in the infusion used in our study.

Before addressing behavioral studies, it is important to highlight that these were carried out based on evidence establishing a relationship between nonalcoholic fatty liver disease (NAFLD), anxiety, and depression [76]. Our study, although primarily focused on investigating NAFLD under treatment with *Moringa*, considered the possibility of comparing potential effects on behavioral aspects. These aspects serve to explore potential indirect effects associated with the treatment. In the first place, the detection of alkaloids, for instance, within the extract provides valuable insights into the chemical composition of *Moringa oleifera*. Alkaloids are known to have diverse physiological effects, including potential impacts on behavior. Our results showed that the group that received MO infusion showed higher mobility than the SD diet group and the ethanolic extract group. Regarding anxiety-like behavior, none of the study groups qualified for anxious behavior. This result may suggest that although the group that consumed the MO infusion increased their mobility, they did not present behaviors related to anxiety-like behavior. Previous studies have suggested that MO has protective effects on behavioral issues. For example, in the study by Islam et al. 2020 [25], effects of MO were found in the central nervous system for the control of anxiety-related disorders, and the administration of MO was found to be potentially an effective stress reliever [77, 78]. In fact, the direct relevance of these behavioral studies to NAFLD might be limited, and their inclusion contributes to a holistic understanding of the treatment's effects. It is possible that these behavioral changes, although subtle, could indirectly influence factors such as food consumption or physical activity, which in turn could affect the metabolic parameters under investigation, such as hyperlipidemia and hyperglycemia, but more studies are needed. A strength of the study is the simultaneous analyses of various therapeutic effects of *M. oleifera* when it is used in individuals who already have established NAFLD. Its preventive effect was not analyzed but rather its ability to reverse various deleterious effects caused by the disease. Likewise, the effect of the ethanolic extract and the hot MO infusion was analyzed, which offered information on two therapeutic presentations, which can be used in the future, according to the patient's needs or feasibility of use. One of the perspectives for the present study is to evaluate both the *M. oleifera* infusion and the ethanolic extract in mice on a standard diet or under other diets such as a ketogenic diet to determine the presence or absence of possible protective/toxic effects. The authors are aware that this study has several limitations since the molecular mechanisms of the therapeutic effects found were not analyzed. Likewise, the phytochemical study of the species is not ruled out as a future possibility and should be explored to clarify the possible molecular mechanisms responsible for its biological activity.

Furthermore, future studies will aim to investigate the impact of *M. oleifera* on inflammatory cytokines, particularly those affecting insulin signaling. In addition, the effect of *M. oleifera* treatment on reactive oxygen species (ROS) and certain activities must be explored to better understand its potential role in addressing insulin resistance and hepatic steatosis. As a limitation, it is important to note that the current study did not examine the expression of SGOT and SGPT at the levels of mRNA or protein, nor were any functional assays completed. Understanding the impact of *M. oleifera* on these specific markers would provide valuable insights into its comprehensive effects on liver function. Future investigations should consider incorporating these analyses to further enhance the understanding of the mechanisms underlying the observed outcomes.

Despite these limitations, the current study provided evidence for the effects of MO on nonalcoholic fatty liver, hyperlipidemia, and hyperglycemia in an HFD mouse model, which may contribute to knowledge and future studies performed in patients with these diseases.

## 5. Conclusion

In conclusion, chronic oral administration of MO infusion leaf extract demonstrates a therapeutic impact, improving liver histology and biochemical markers in a murine NAFLD model. Furthermore, the therapeutic effectiveness of MO extract is notably enhanced when administered as an infusion compared to the ethanolic extract in the same NAFLD murine model. Further investigations are warranted to elucidate the underlying mechanisms driving these observed effects.

## Figures and Tables

**Table 1:** Phytochemical screening components of *M. oleifera* ethanolic extract and infusion.

Phytochemical compounds	MO ethanolic extract	MO infusion tea
Tannins	+	−
Flavonoids	−	+
Steroids	+	+
Alkaloids	+++	+
Saponins	+	++
TFC^a,^^*∗*^	QE = 19.40 ± 10.68 *μ*g/mg extract	QE = 172.66 ± 7.58 *μ*g/mg extract
FRPA^b,^^*∗*^	% age reduction = 102.62 ± 5.30	% age reduction = 92.25 ± 3.6
TAC^c,^^*∗*^	% age TAC = 65.57 ± 1.27	% age TAC = 56.62 ± 0.42

+++ = appreciable amount (positive within 5 mins); ++ = moderate amount (positive after 5 mins but within 10 mins); + = trace amount (positive after 10 mins but within 15 mins); − = completely absent. ^a^TFC: expressed in quercetin equivalents (QE) *μ*g/mg of extract. ^b^FRPA: expressed as % reducing power relative to the ascorbic acid control. ^c^TAC: expressed as % antioxidant capacity relative to the ascorbic acid control. ^*∗*^Concentration at 5 mg/ml.

**Table 2:** Biochemical and hematic parameters according to the groups studied.

	Parameters	Healthy (*n* = 11)	NAFLD-placebo (*n* = 11)	NAFLD-MO ethanolic extract (*n* = 11)	NAFLD-MO infusion (*n* = 11)	*p* ANOVA
Biochemical	Cholesterol (mg/dL)	116.9 ± 12.0	181.2 ± 15.0^a^	120.1 ± 11.8^d^	104.3 ± 11.4^e^	<0.001
	Triglycerides (mg/dL)	114.8 ± 15.5	185.5 ± 12.6^a^	116.6 ± 15.7^d^	93.8 ± 11.2^ef^	<0.001
	Total lipids (mg/dL)	156.1 ± 9.6	299.8 ± 15.1^a^	199.3 ± 14.1^d^	184.3 ± 13.0^e^	<0.001
	SGOT (U/L)	184.0 ± 14.2	403.0 ± 12.1^a^	210.1 ± 14.3^d^	178.1 ± 15.0^ef^	<0.001
	SGPT (U/L)	128.9 ± 11.9	241.6 ± 16.3^a^	130.8 ± 13.9^d^	113.2 ± 7.0^ef^	<0.001
	Blood glucose (mg/dL)	88.9 ± 13.8	141.1 ± 20.9^a^	109.0 ± 9.2^d^	114.1 ± 14.8^e^	0.002
	Urea (mg/dL)	11.9 ± 5.3	15.4 ± 5.8	12.8 ± 2.8	11.7 ± 5.1	0.525

Hematic	Hemoglobin (g/dL)	12.5 ± 1.1	14.3 ± 0.5^a^	14.18 ± 0.2	14.2 ± 0.7^c^	<0.001
	Hematocrit (%)	35.6 ± 4.1	39.7 ± 1.5	37.77 ± 0.1	40.0 ± 0.74	0.902
	Platelets (10^∧^3/*μ*L)	1207.2 ± 38.1	1210.8 ± 48.8	1008.7 ± 54.1	1127.1 ± 31.5	0.575
	Leukocytes (10^∧^9/*μ*L	6.4 ± 2.0	6.4 ± 2.1	6.4 ± 1.6	6.45 ± 0.6	0.262
	Erythrocytes (10^∧^12/*μ*L)	8.1 ± 1.0	7.7 ± 0.9	7.6 ± 0.2	7.80 ± 0.3	0.675

All values are expressed as mean ± SEM; the differences of Tukey's post hoc analysis are marked when they are significant: ^a^*p* < 0.05, healthy vs. NAFLD-placebo, ^b^*p* < 0.05, healthy vs. NAFLD-MO ethanolic extract, ^c^*p* < 0.05, healthy vs. NAFLD-MO infusion, ^d^*p* < 0.05, NAFLD-placebo vs. NAFLD-MO ethanolic extract, ^e^*p* < 0.05, NAFLD-placebo vs. NAFLD-MO infusion, and ^f^*p* < 0.05, NAFLD-MO ethanolic extract vs. NAFLD-MO infusion (*n* = 11 per group).

**Table 3:** Fasting glucose levels (mg/dL) at various times during NAFLD model generation.

Glucose	Healthy (*n* = 11)	NAFLD model (*n* = 33)	*p* Tukey's test
Initial	84.80 ± 0.8	88.60 ± 3.3	0.155
3 months	94.00 ± 0.7	99.02 ± 2^a^	**<0.001**
6 months	96.70 ± 2.4	138.00 ± 2.0^a^	**<0.001**

All values are expressed as mean ± SEM; the differences of Tukey's post hoc analysis are marked when they are significant: ^a^*p* < 0.05, when compared healthy vs. NAFLD-placebo (*n* = 11 per group).

**Table 4:** Organ weight of the experimental groups.

Organ weight (g)	Healthy (*n* = 11)	NAFLD-placebo (*n* = 11)	NAFLD-MO ethanolic extract (*n* = 11)	NAFLD-MO infusion (*n* = 11)	*p*ANOVA
Liver	1263.2 ± 347.7	1531.4 ± 298.3	1379.5 ± 379.6	1174.5 ± 160.6^e^	0.002
Right kidney	0.16 ±0 .03	0.20 ±0 .10	0.19 ± 0.01	0.19 ±0 .02	0.124
Left kidney	0.17 ±0 .03	0.20 ±0 .03	0.19 ± 0.04	0.19 ±0 .04	0.340
Pancreas	125.0 ± 40.0	157.0 ± 68.5	128.5 ± 35.6	139.5 ± 33.9	0.506
Brain	420.6 ±0 .36	414.3 ± 19.8	400.8 ± 28.2	408.7 ±0 .42.9	0.833
Ovaries	219.9 ± 69.6	232.3 ±0 .57.7	234.9 ± 108.3	236.6 ± 36.0	0.154
Epididymal fat^*∗*^	133.2 ± 18.8	303.4 ± 106.4^a^	214.8 ± 22.9^b d^	207.5 ± 33.9^ec^	<0.001

All values are expressed as mean ± SEM; the differences of Tukey's post hoc analysis are marked when they are significant: ^a^*p* ≤ 0.05, when compared healthy vs. NAFLD-placebo, ^b^*p* ≤ 0.05, healthy vs. NAFLD-MO ethanolic extract, ^c^*p* ≤ 0.05, healthy vs. NAFLD-MO infusion, ^d^*p* ≤ 0.05, NAFLD-placebo vs. NAFLD-MO ethanolic extract, ^e^*p* ≤ 0.05, NAFLD-placebo vs. NAFLD-MO infusion, (*n* = 11 per group). ^*∗*^Only for male mice in each group (healthy group (*n* = 6) and NAFLD-placebo and experimental groups (*n* = 6)).

**Table 5:** Histopathological parameters according to the groups studied.

Parameters	Healthy (*n* = 11)	NAFLD-placebo(*n* = 11)	NAFLD-MO ethanolic extract (*n* = 11)		NAFLD-MO infusion (*n* = 11)	*p* ANOVA
% steatosis	5.00 ± 3.3	68.18 ± 10.5^a^	20.41 ± 8.5^b^	14.1 ± 3.1^c,d^		<0.001
% inflammation	0 (0-1)	3 (2-3)^a^	2 (1-2)^b^	1 (1-2)^c,d^		<0.001

All values are expressed as mean ± SEM, the differences of Tukey's post hoc analysis, Kruskal–Wallis, and Wilcoxon are marked when they are significant: ^a^*p* ≤ 0.05 when compared healthy vs. NAFLD-placebo, ^b^*p* ≤ 0.05, NAFLD-placebo vs. NAFLD-MO ethanolic extract, ^c^*p* ≤ 0.05, NAFLD-placebo vs. NAFLD-MO infusion, and ^d^*p* ≤ 0.05, NAFLD-MO ethanolic extract vs. NAFLD-MO infusion (*n* = 11 per group). 0: none, 1: mild, 2: moderate, and 3: sever.

**Table 6:** Locomotor activity evaluation according to the groups studied.

Parameters	Healthy (*n* = 11)	NAFLD-placebo(*n* = 11)	NAFLD-MO ethanolic extract (*n* = 11)	NAFLD-MO infusion (*n* = 11)	*p* ANOVA
Number of rotations	8.85 ± 2.5	9.1 ± 3.0	12.58 ± 4.4	15.00 ± 4.7^b,d^	0.003
Number of falls	17.07 ± 3.7^a^	21.91 ± 3.1	16.58 ± 3.2^c^	16.25 ± 2.2^d^	<0.001
Anxiety-like behavior index	0.86 ± 0.09	0.86 ± 0.11	0.84 ± 0.18	0.68 ± 0.18^b,d,e^	<0.001

All values are expressed as mean ± SEM; the differences of Tukey's post hoc analysis are marked when they are significant: ^a^*p* ≤ 0.05, healthy vs. NAFLD-placebo, ^b^*p* ≤ 0.05, healthy vs. NAFLD-MO infusion, ^c^*p* ≤ 0.05, NAFLD-placebo vs. NAFLD-MO ethanolic extract, ^d^*p* ≤ 0.05, NAFLD-placebo vs. NAFLD-MO infusion, and ^e^*p* ≤ 0.05, NAFLD-MO ethanolic extract vs. NAFLD-MO infusion (*n* = 11 per group).

## Data Availability

The datasets used and/or analyzed during the current study are available from the corresponding author on reasonable request.

## Supplementary Materials

Supplementary Materials S1 Table: comparisons of chemical shifts of the MO extract and infusion vs. some of the principal metabolites isolated from the leaves of *Moringa oleifera* in CDCl_3_. SII Table: food intake and weight of the experimental groups. SIII Table: glucose tolerance curve according to the groups studied. S1 Figure: ^1^H NMR spectra for the infusion leaves of *Moringa oleifera*, ^1^H-NMR in CDCl_3_ (400 MHz). S2 Figure: ^1^H NMR spectra for the ethanolic extract from the leaves of *Moringa oleifera*, ^1^H-NMR in CDCl_3_ (400 MHz). S3 Figure: ^13^C NMR spectra for the infusion leaves of *Moringa oleifera*, ^13^C-NMR in CDCl_3_ (100 MHz). S4 Figure: ^13^C NMR spectra for the ethanolic extract from the leaves of *Moringa oleifer*a, ^13^C-NMR in CDCl_3_ (100 MHz).
