# Supplementary material for: Efficacy of Hot Tea Infusion vs. Ethanolic Extract of Moringa oleifera for the Simultaneous Treatment of Nonalcoholic Fatty Liver, Hyperlipidemia, and Hyperglycemia in a Murine Model Fed with a High-Fat Diet
Source: J Nutr Metab. 2024 Feb 12;2024:2209581. doi: 10.1155/2024/2209581 (PMC10876314; doi:10.1155/2024/2209581)
Supplement: Supplementary Materials — S1 Table: comparisons of chemical shifts of the MO extract and infusion vs. some of the principal metabolites isolated from the leaves of Moringa oleifera in CDCl3. SII Table: food intake and weight of the experimental groups. SIII Table: glucose tolerance curve according to the groups studied. S1 Figure: 1H NMR spectra for the infusion leaves of Moringa oleifera, 1H-NMR in CDCl3 (400 MHz). S2 Figure: 1H NMR spectra for the ethanolic extract from the leaves of Moringa oleifera, 1H-NMR in CDCl3 (400 MHz). S3 Figure: 13C NMR spectra for the infusion leaves of Moringa oleifera, 13C-NMR in CDCl3 (100 MHz). S4 Figure: 13C NMR spectra for the ethanolic extract from the leaves of Moringa oleifera, 13C-NMR in CDCl3 (100 MHz). [file 2209581.f1.zip › 2209581.f1.docx]

**SI Table.** **Comparisions of chemical shifts of the MO extract and infusion vs some of the principal metabolites isolated from the leaves of *Moringa oleifera* in CDCl_3_**

| **Signal** | **MO extract ^1^H**  **(A)** | | **MOinfusion**  **(B)** | | **Niazirin ^X^** | | **Niazirinin ^Y^** | | **4-{(4’ -0-Acetyl-a-L-rhamnosyloxy) benzyl) isothiocyanate ^X^** | | **β-sitosterol ^Z^** | |
| --- | --- | --- | --- | --- | --- | --- | --- | --- | --- | --- | --- | --- |
| **δ** | **^1^H** | **^13^C** | **^1^H** | **^13^C** | **^1^H** | **^13^C** | **^1^H** | **^13^C** | **^1^H** | **^13^C** | **^1^H** | **^13^C** |
| 1 | 1.25 s | 14.24 | 0.86 m | 14.23 | 1.27 d(6.2) | 17.49 | 1.19d | 17.46 | 1.19 d (6.2) | 17.42 | 0.86-1.10 (5x3H) | 11.9 |
| 2 | 1.60 s | 16.16 | 9.98 t | 14.40 | 3.54 t (9.41) | 22.9 | 3.58qd | 21.05 | 3.87 qd (9.4, 6.2) | 20.87 | 3.53 m | 12.1 |
| 3 | 1.75 d | 17.82 | 1.25 br.s | 16.14 | 3.69 s | 68.83 | 3.68s | 22.91 | 4.09 dd (9.4,3.5) | 47.5 | 5.35 m | 18.9 |
| 4 | 2.05 br.s | 22.84 | 1.60 s | 17.81 | 3.75 m | 70.87 | 4.09 dd(9.6, 3.5) | 66.36 | 4.14 dd (3.5, 1.6) | 66.99 |  | 19.1 |
| 5 | 3.68 s | 23.50 | 1.68 s | 20.70 | 3.97 dd(9.1, 3.4) | 71.7 | 4.15 dd(3.5, 1.6) | 70.13 | 4.64s | 67.94 |  | 19.5 |
| 6 | 3.69 s | 25.83 | 2.04 br.s | 22.56 | 4.14 dd(3.4, 1.9) | 73.48 | 4.87 t(9.6) | 70.65 | 4.85 t (9.4) | 70.01 |  | 19.9 |
| 7 | 4.08 br.s | 26.57 | 2.81 t | 22.83 | 5.51 d(1.9) | 97.96 | 5.55 d (1.6) | 75.49 | 5.55 d(1.6) | 73.48 |  | 21.2 |
| 8 | 4.10 br.s | 26.85 | 3.23 s | 23.56 | 7.05 d (8.9) | 116.65 | 7.05 d (8.7) | 97.19 | 7.07 d (8.08) | 98 |  | 23.1 |
| 9 | 4.15 br.s | 26.94 | 3.40 s | 25.79 | 7.25 d (8.5) | 123.72 | 7.24 d (8.7) | 116.87 | 7.25 d (8.8) | 116.8 |  | 24.4 |
| 10 | 5.11 br.s | 29.50 | 3.69 s | 26.57 |  | 129.23 |  | 123.7 |  | 128.6 |  | 26.2 |
| 11 | 5.37 br.s | 29.75 | 3.88 s | 26.81 |  | 132.64 |  | 129.25 |  | 128.81 |  | 28.3 |
| 12 | 7.00 s | 29.84 | 4.10 d | 26.86 |  | 156.03 |  | 131.7 |  | 131.68 |  | 29.2 |
| 13 | 7.52 s | 32.08 | 4.58 m | 26.95 |  |  |  | 156 |  | 155.65 |  | 31.7 |
| 14 | 7.53 s | 32.15 | 5.10 br.s | 29.50 |  |  |  | 172.01 |  | 168.98 |  | 31.9 |
| 15 | 7.53 s | 32.38 | 5.38 m | 32.16 |  |  |  |  |  |  |  | 32 |
| 16 | 7.54 s | 39.89 | 6.63 s | 39.88 |  |  |  |  |  |  |  | 34 |
| 17 | 7.70 s | 59.19 | 6.41 s | 59.18 |  |  |  |  |  |  |  | 36.2 |
| 18 | 7.71s | 76.91 | 7.96 s | 124.33 |  |  |  |  |  |  |  | 36.6 |
| 19 | 7.71 s | 77.23 | 7.99s | 124.45 |  |  |  |  |  |  |  | 37.3 |
| 20 | 7.72 s | 77.37 | 8.00 s | 124.60 |  |  |  |  |  |  |  | 39.8 |
| 21 | 8.00 s | 124.32 | 8.03s | 124.69 |  |  |  |  |  |  |  | 42.3 |
| 22 | 8.55 d | 124.45-125.20 | 8.05s | 125.12 |  |  |  |  |  |  |  | 42.4, 46 |
| 23 | 9.40 s | 127.29-128.48 | 8.56d | 125.20-128.47 |  |  |  |  |  |  |  | 50.2 |
| 24 | 9.45 br.s | 130.40 | 9.39s | 130.39-130.43 |  |  |  |  |  |  |  | 56.1 |
| 25 | 9.50 s | 131.39-132.13 | 9.52s |  |  |  |  |  |  |  |  | 56.8 |
| 26 | 9.54 s | 135.05-135.53 | 9.68s | 131.35-132.12 |  |  |  |  |  |  |  | 71.9 |
| 27 | 9.63 s | 140.09 | 10.41s | 135.04-136.21 |  |  |  |  |  |  |  | 121.8 |
| 28 | 9.61 s | 200.58 | 11.17s | 140.02 |  |  |  |  |  |  |  | 140.8 |
| **^a^**δ from TMS (ppm). ^1^H NMR [400 MHz, CDCl_3_, J (Hz)] and ^13^C NMR (100 MHz) data for MO extract (**A**), and infusion (**B**). br (broad signal). Chemical shifts obtained from x (40,41), Y (40,41) and Z (42). All the NMR data were obtained from similar NMR experiments with CDCl_3_ as a solvent. | | | | | | | | | | | | |


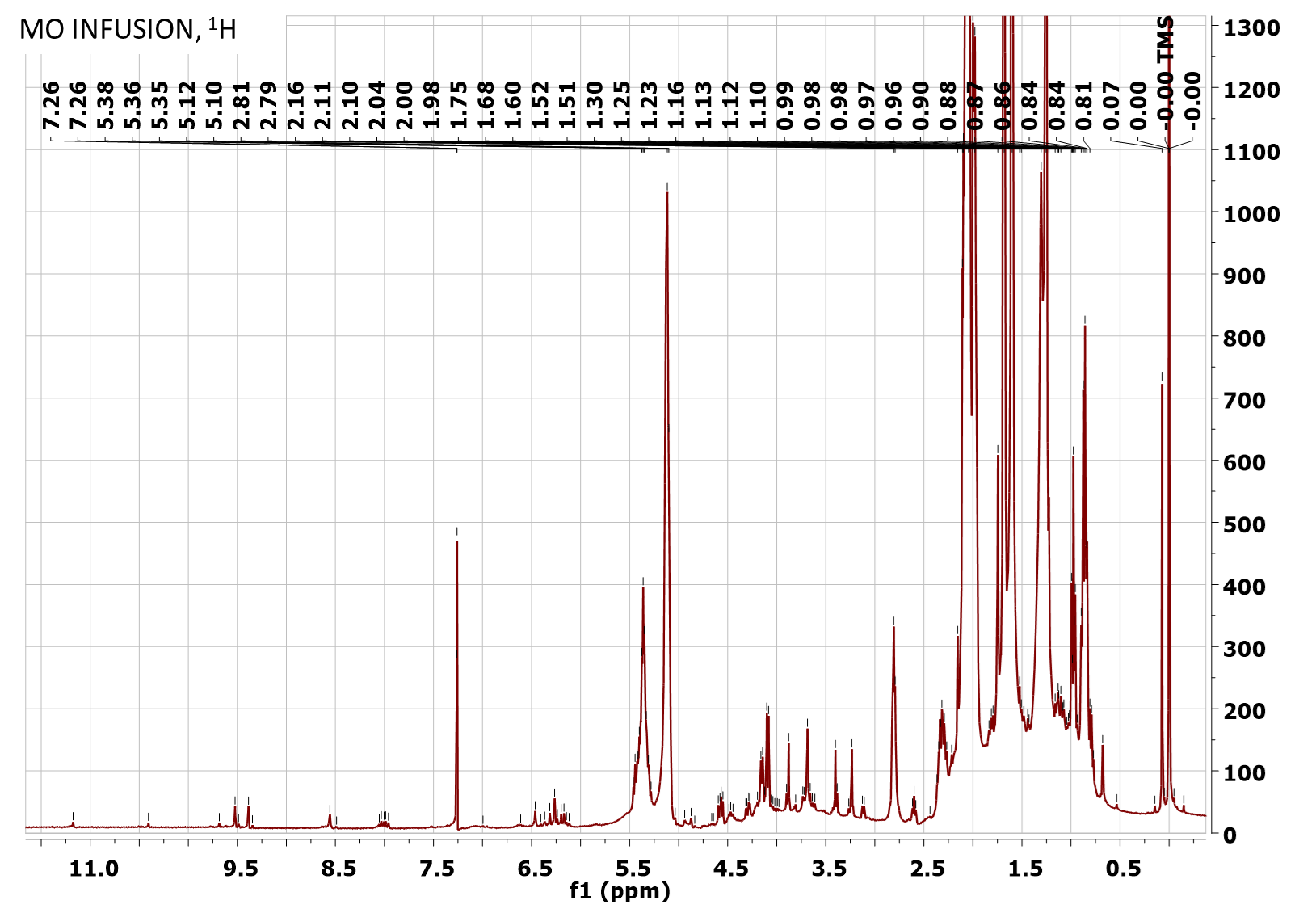


S1 Fig. ^1^H NMR spectra for the infusion leaves of *Moringa oleifera,* ^1^H-NMR in CDCl_3_ (400 MHz)


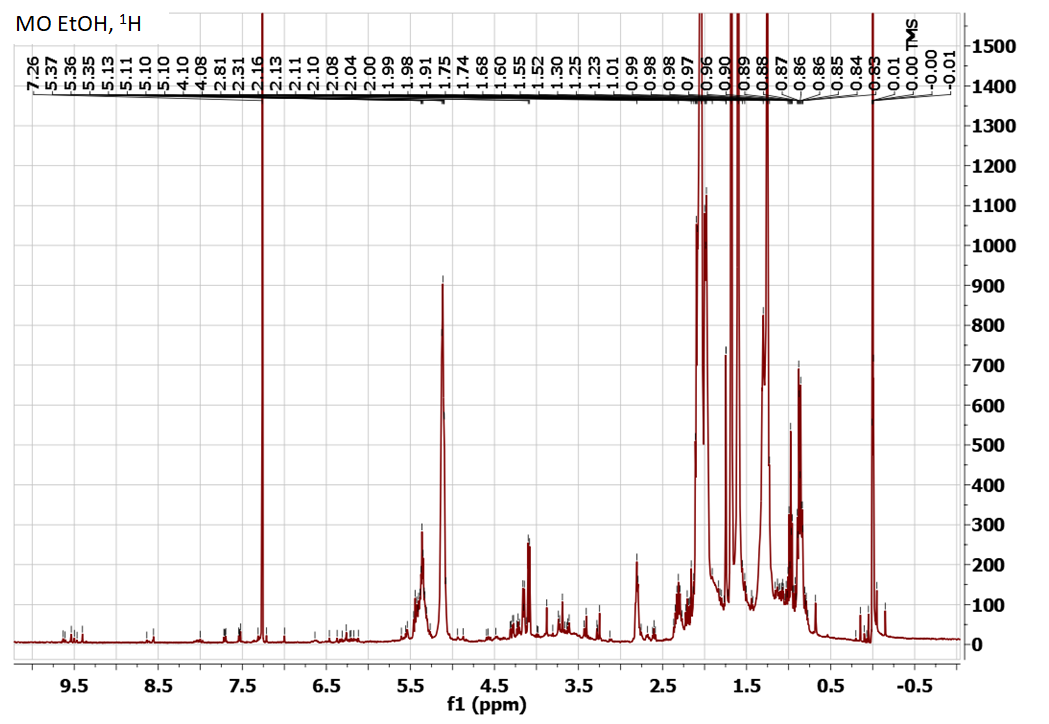


S2 Fig. ^1^H NMR spectra for the ethanolic extract from the leaves of *Moringa oleifera*, ^1^H-NMR in CDCl_3_ (400 MHz)

S3 Fig. ^13^C NMR spectra for the infusion leaves of *Moringa oleifera,* ^13^C-NMR in CDCl_3_ (100 MHz)


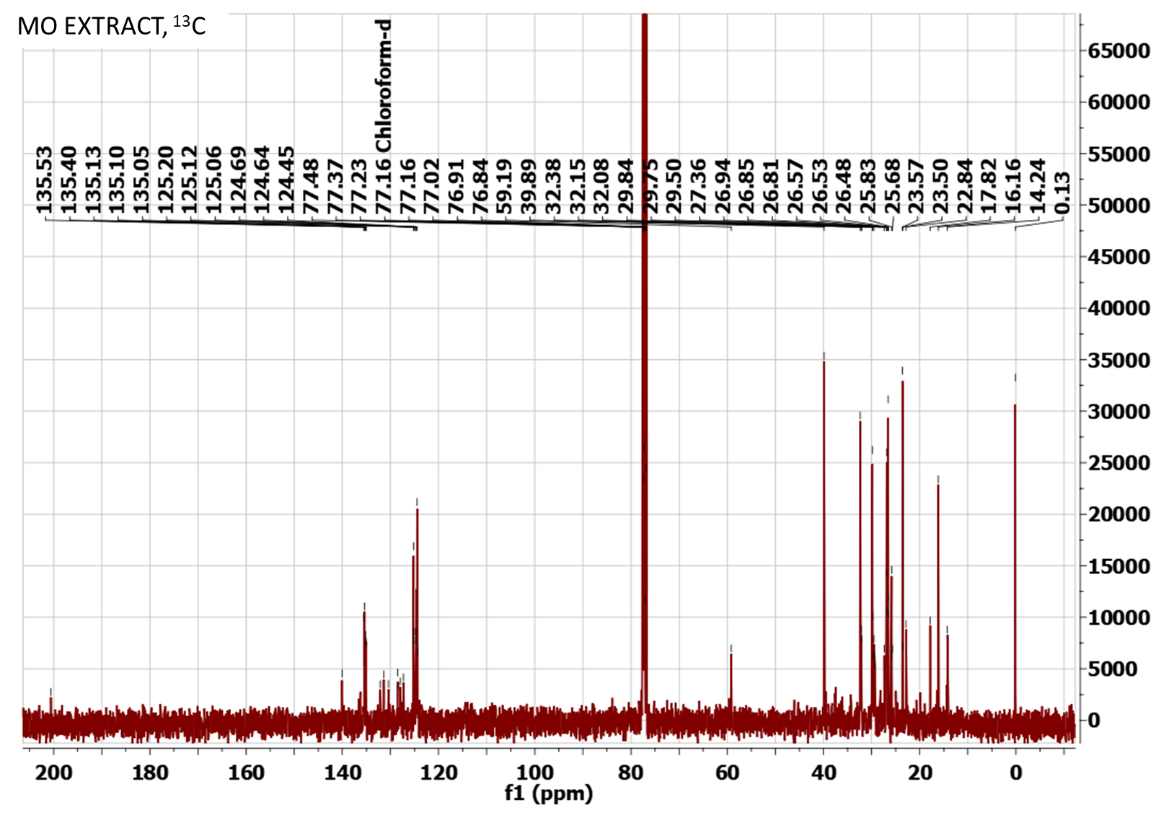


S4 Fig. ^13^C NMR spectra for the ethanolic extract from the leaves of *Moringa oleifera,* ^13^C-NMR in CDCl_3_ (100 MHz)

**SII Table. Food Intake and weight of the experimental groups**

| Month | | Healthy | NAFLD model genesis | NAFLD-placebo | NAFLD-MO ethanolic extract | NAFLD-MO infusion | *p* |
| --- | --- | --- | --- | --- | --- | --- | --- |
| 0 | | 24.57±1.9 | 24.73±1.8 |  |  |  | 0.944 |
| Weight (g) | 1 | 28.36±2.4 | 29.75±2.0 |  |  |  | 0.310 |
|  | 2 | 28.51±2.8 | 30.41±2.1 |  |  |  | 0.061 |
|  | 3 | 28.61±2.8 | 30.77±2.0^a^ |  |  |  | **<0.001**** |
|  | 4 | 28.83±2.6 | 31.19±2.3^a^ |  |  |  | **<0.001**** |
|  | 5 | 29.18±2.3 | 31.57±2.5^a^ |  |  |  | **<0.001**** |
|  | 6 | 29.34±2.2 | 31.85±3.0^a^ |  |  |  | **<0.001**** |
|  | 7 | 29.51±2.3 |  | 33.98±2.1^b^ | 32.12±1.9 | 30.21±2.3 | **<0.001*** |
|  | 8 | 29.95±2.2 |  | 36.92±2.1^b^ | 35.53±1.9 | 25.70±1.2 ^c d e^ | **<0.001*** |
| 0 | | 2.90±1.0 | 3.00±0.9 |  |  |  | 0.978 |
| Food intake (g) | 1 | 3.43±1.4 | 5.40±1.0^a^ |  |  |  | **<0.001**** |
|  | 2 | 4.02±1.0 | 6.24±0.4^a^ |  |  |  | **<0.001**** |
|  | 3 | 4.67±1.8 | 7.02±0.5^a^ |  |  |  | **<0.001**** |
|  | 4 | 5.32±0.6 | 7.40±0.3^a^ |  |  |  | **<0.001**** |
|  | 5 | 5.87±1.3 | 7.64±0.4^a^ |  |  |  | **<0.001**** |
|  | 6 | 6.17±1.2 | 8.15±0.2^a^ |  |  |  | **<0.001**** |
|  | 7 | 6.49±2.0 |  | 9.5±1.1^b^ | 8.11±1.2 | 6.66±1.3 | **<0.001*** |
|  | 8 | 7.54±1.2 |  | 11.0±2.9 ^b^ | 10.27±1.4 | 5.64±2.0 ^d e^ | **<0.001*** |

All values are expressed as mean ± SEM, *the differences of the Tukey`s post hoc analysis are marked when they are significant in four groups, **the differences of the Tukey`s post hoc analysis are marked when they are significant: (a) P≤0.05, healthy vs NAFLD model genesis, (b) P≤0.05, healthy vs NAFLD-placebo, (c) P≤0.05, healthy vs NAFLD-MO infusion, (d) P≤0.05, NAFLD-placebo vs NAFLD-MO infusion, (e) P≤0.05, NAFLD-MO extract vs NAFLD-MO infusion. The comparisons between NAFLD-placebo vs NAFLD-MO extract and healthy vs NAFLD-MO extract did not show statistically significant differences at a significance level of P≤0.05.

**SIII Table. Glucose tolerance curve according to the groups studied**

| Minutes | | Healthy | NAFLD-placebo | NAFLD-MO ethanolic  extract | NAFLD-MO infusion | *p* ANOVA |
| --- | --- | --- | --- | --- | --- | --- |
| Baseline | 0 | 96.70±2.4 | 138.00±2.0^a^ | 130.00±2.6^b^ | 135.50±2.6^c^ | **<0.001** |
|  | 30 | 157.60±2.9 | 336.00±2.6^a^ | 329.33±3.0 ^b^ | 335.75±2.2^c^ | **<0.001** |
|  | 60 | 118.40±3.2 | 241.00±3.6^a^ | 234.66±1.5 ^b^ | 241.75±2.8 ^c^ | **<0.001** |
|  | 90 | 103.40±3.2 | 211.00±3.6 ^a^ | 205.00±1.0 ^b^ | 209.75±2.6 ^c^ | **<0.001** |
|  | 120 | 93.50±3.3 | 216.00±3.6 ^a^ | 210.66±1.1^b^ | 216.00±2.7 ^c^ | **<0.001** |
| After treatment | 0 | 99.57±3.1 | 178.09±3.9 ^a^ | 107.41±3.7^b,d^ | 166.75±3.7^c,f^ | **<0.001** |
|  | 30 | 159.57±3.1 | 377.81±3.9^a^ | 309.33±3.9^b,d^ | 366.75±3.7 ^c,f^ | **<0.001** |
|  | 60 | 117.71±3.0 | 277.27±3.9 ^a^ | 205.75±3.8^b,d^ | 270.33±3.1^c,f^ | **<0.001** |
|  | 90 | 102.57±3.1 | 217.18±3.9 ^a^ | 136.50±3.7^b,d^ | 225.41±3.6^c,f^ | **<0.001** |
|  | 120 | 95.57±3.1 | 224.90±3.3 ^a^ | 103.83±3.1 ^d^ | 218.00±3.3^c,f^ | **<0.001** |
| All values are expressed as mean ± SEM, differences from Tukey`s post hoc analysis are marked when they are significant: ^a^*p* <0.05, healthy vs. NAFLD-placebo, ^b^ *p* <0.05, healthy vs. NAFLD-MO ethanolic extract, ^c^ *p* <0.05, healthy vs. NAFLD-MO infusion, ^d^ *p* <0.05, NAFLD-placebo vs. NAFLD-MO ethanolic extract, ^e^ *p* <0.05, NAFLD-MO ethanolic extract vs. NAFLD-MO infusion. n= 11 per group. | | | | | | |
